# Menstrual Cycle Variations in Gray Matter Volume, White Matter Volume and Functional Connectivity: Critical Impact on Parietal Lobe

**DOI:** 10.3389/fnins.2020.594588

**Published:** 2020-12-22

**Authors:** Timothy J. Meeker, Dieuwke S. Veldhuijzen, Michael L. Keaser, Rao P. Gullapalli, Joel D. Greenspan

**Affiliations:** ^1^Department of Neurosurgery, Johns Hopkins University, Baltimore, MD, United States; ^2^Department of Neural and Pain Sciences, University of Maryland School of Dentistry, Baltimore, MD, United States; ^3^Center to Advance Chronic Pain Research, University of Maryland Baltimore, Baltimore, MD, United States; ^4^Institute of Psychology, Health, Medical and Neuropsychology Unit, Leiden University, Leiden, Netherlands; ^5^Leiden Institute for Brain and Cognition, Leiden, Netherlands; ^6^Department of Diagnostic Radiology and Nuclear Imaging, University of Maryland School of Medicine, Baltimore, MD, United States

**Keywords:** dorsal attention network, inferior parietal lobule, menstrual cycle, cortical thickness, resting state functional connectivity, somatosensory sensitivity, pain sensitivity

## Abstract

The role of gonadal hormones in neural plasticity remains unclear. This study aimed to examine the effects of naturally fluctuating hormone levels over the menstrual cycle in healthy females. Gray matter, functional connectivity (FC) and white matter changes over the cycle were assessed by using functional magnetic resonance imaging (fMRI), resting state fMRI, and structural MRIs, respectively, and associated with serum gonadal hormone levels. Moreover, electrocutaneous sensitivity was evaluated in 14 women in four phases of their menstrual cycle (menstrual, follicular, ovulatory, and luteal). Electrocutaneous sensitivity was greater during follicular compared to menstrual phase. Additionally, pain unpleasantness was lower in follicular phase than other phases while pain intensity ratings did not change over the cycle. Significant variations in cycle phase effects on gray matter volume were found in the left inferior parietal lobule (IPL) using voxel-based morphometry. Subsequent Freesurfer analysis revealed greater thickness of left IPL during the menstrual phase when compared to other phases. Also, white matter volume fluctuated across phases in left IPL. Blood estradiol was positively correlated with white matter volume both in left parietal cortex and whole cortex. Seed-driven FC between left IPL and right secondary visual cortex was enhanced during ovulatory phase. A seed placed in right IPL revealed enhanced FC between left and right IPL during the ovulatory phase. Additionally, we found that somatosensory cortical gray matter was thinner during follicular compared to menstrual phase. We discuss these results in the context of likely evolutionary pressures selecting for enhanced perceptual sensitivity across modalities specifically during ovulation.

## Introduction

Studies of gray matter in the female brain across the natural menstrual cycle have revealed phase effects in several brain areas including the hippocampus, fusiform gyrus, amygdala, and frontal and parietal cortices (Protopopescu et al., 2008; Pletzer et al., 2010, 2015, 2018; Hagemann et al., 2011; Lisofsky et al., 2015; De Bondt et al., 2016; Pletzer, 2019). Relatedly, several studies have reported that estradiol enhances performance on hippocampal-dependent and prefrontal cortex-dependent tasks in female rats, mice, non-human primates as well as pre- and post-menopausal humans (Berman et al., 1997; Daniel et al., 1997; Luine et al., 1998; Korol and Kolo, 2002; Lacreuse et al., 2002; Rapp et al., 2003; Li et al., 2004; Sandstrom and Williams, 2004; Luine, 2008; Spencer et al., 2008; Bayer et al., 2015, 2018; Jacobs et al., 2016; Wei et al., 2018). With accurate assessment of hormone levels and confirmed ovulation, the menstrual cycle phase enables the study of estrogens and progesterone on neurophysiology and cognitive function (Greenspan et al., 2007; Sundstrom Poromaa and Gingnell, 2014).

Menstrual cycle phase effects on sensory perception have been demonstrated for over 80 years (Herren, 1933; Kenshalo, 1966). Many studies have reported heightened sensitivity during follicular or ovulatory phases compared to menstrual or luteal phases including visual, auditory, olfactory, and somatosensory stimuli (Diespecker and Kolokotronis, 1971; Diamond et al., 1972; Friedman and Meares, 1978, 1979; Parlee, 1983; Symons et al., 1990; Hummel et al., 1991; Guttridge, 1994; Caruso et al., 2001, 2003; Grillo et al., 2001; Navarrete-Palacios et al., 2003; Derntl et al., 2013; Renfro and Hoffmann, 2013; Veldhuijzen et al., 2013; Alves et al., 2017). Significant consistent variation in perceptual sensitivity to several modalities of sensory stimulation across the menstrual cycle, particularly during phases with high estrogens suggests an underlying neurophysiological mechanism in multimodal sensory cortex.

The effects of ovarian hormones on cortical excitability measured using transcranial magnetic stimulation include increased cortical inhibition in luteal compared to follicular phase and increased excitability associated with late compared to early follicular or luteal phase (Smith et al., 1999, 2002; Inghilleri et al., 2004; Hausmann et al., 2006; Hattemer et al., 2007). Furthermore, the amplitude of visual evoked potentials (VEPs) to sexually salient stimuli are greatest during ovulatory phase, while effects of menstrual cycle on VEPs to neutral stimuli are mixed (Johnston and Wang, 1991; Yilmaz et al., 1998; Tasman et al., 1999; Krug et al., 2000; Solis-Ortiz et al., 2004; Avitabile et al., 2007; Brotzner et al., 2015). Several studies report inhibition of alpha EEG power and driving responses to visual stimuli during late follicular and ovulatory phase compared to augmentation of alpha measures during luteal phase (Vogel et al., 1971; Becker et al., 1982; Solis-Ortiz et al., 1994; Kaneda et al., 1997; Brotzner et al., 2014; Bazanova et al., 2017).

Studies of resting state fMRI functional connectivity (FC) provide important information on brain network function (Buckner and Vincent, 2007). While previous studies have investigated the menstrual cycle phase effect on changes in FC networks, most have focused on the hippocampus or found no effects (Hjelmervik et al., 2014; De Bondt et al., 2015; Syan et al., 2017). Findings include reports of enhanced FC between the hippocampus and bilateral superior parietal lobules during late follicular phase (pre-ovulatory) and that regions in the frontal and parietal cortex significantly change in FC with the default mode network (Petersen et al., 2014; Lisofsky et al., 2015). Much work remains to unravel the complex effects of the menstrual cycle phase on brain structure and function.

In the present study, gray and white matter morphology and functional connectivity across the menstrual cycle was examined. We hypothesized significant variation in gray and white matter volume measures across the menstrual cycle in healthy females. Given significant cycle phase effects on morphometric measures, we additionally determined effects of estrogen and progesterone concentration at the time of the scan and correlated these with cortical thickness and white matter volume. Additionally, we evaluated menstrual cycle effects on cortical thickness in the bilateral somatosensory cortex as demonstrated in previously reported menstrual cycle effect on tactile, thermal, and pressure pain sensitivity (Herren, 1933; Veldhuijzen et al., 2013; Alves et al., 2017). We complement these brain morphometric results by showing the effect of menstrual cycle phase on cutaneous electrical sensitivity and reports of pain intensity and unpleasantness in response to electrical stimuli. Finally, we hypothesized modulated FC involving those areas with significant cycle variation in gray and white matter volume during the menstrual cycle.

## Materials and Methods

### Participants and Experimental Procedure

Fourteen healthy right-handed women participated in this study. Eligible participants were women aged 18 to 45 who were right-handed and spoke English fluently. Participants reported a normal recurrent menstrual cycle of 25 to 35 days in which ovulation and menstruation took place. This was documented by completion of 2 months of daily menstrual cycle diaries before the start of data collection. Finally, participants were eligible when they were willing and able to undergo MRI scanning. Exclusion criteria included: (1) cognitive impairment that prevented understanding of the consent form or test instructions, which was assessed by the Mini Mental State Examination; (2) health problems as assessed by self-report including history of drug or alcohol abuse, psychiatric disorder or dysfunction requiring treatment, history of abnormal electrocardiogram, pulmonary disease, chronic respiratory disease, hypertension, heart or artery disease including heart failure and stroke, renal disease, seizure disorders, endocrine disorders such as thyroid and diabetes, chronic pain, arthritis, insomnia, reproductive system problems such as endometriosis, carpal tunnel syndrome, undergoing chemotherapy or radiation treatments; (3) obesity (body mass index > 30); (4) if the painful stimulation failed to elicit a rating of 60 on a 0 to 100 visual analog scale (VAS) of pain intensity; (5) unable to undergo MRI scanning; (6) pregnancy; (7) use of psychotropic medications during the preceding 6 months; (8) use of hormone therapy including hormonal birth control pills during the preceding month; (9) use of tobacco in the last 6 months; (10) having experienced any serious injury to the body regions to be tested; or (11) if regularly exercising more than 1 h per day, 3 times per week. Prior to the four planned fMRI scanning sessions, participants filled out daily menstrual cycle diaries for 2 months demonstrating a normal recurrent menstrual cycle of 25 to 35 days duration in which ovulation and menstruation took place. Participants reported self-assessed ovulation using at-home ovulation test kits which detect the presence of luteinizing hormone (LH) in urine. Participants recorded basal body temperature (BBT) upon wakening every morning. After verifying participants completed their first month’s diary, we requested them to maintain this diary for their entire study participation. After demonstrating two regular complete serial menstrual cycles in which ovulation could be detected, each woman participated in four experimental fMRI sessions. These sessions took place during the menstrual phase (within 2 to 4 days of the onset of menses), the midfollicular phase (within 6 to 8 days of onset of menses when estradiol and progesterone levels are low), the periovulatory period (the day of or the day after the first positive ovulation test; about 14 days after onset of the menstrual cycle when estradiol levels are high and progesterone are low), and the midluteal phase (1 week after ovulation; about 20 days after onset of menses when both estradiol and progesterone levels are high) (Veldhuijzen et al., 2013). We counterbalanced participant order of testing by assigning them to have their first experimental session in one of the four phases of the menstrual cycle. The University of Maryland, Baltimore (UMB) Institutional Review Board for the Protection of Human Subjects approved the study. All participants provided written informed consent.

### MRI Recording

We recorded MRI data on a 3-T Tim Trio scanner (Siemens Medical Solutions, Malvern, PA) with a 12-channel head coil with parallel imaging capability. A gradient echo single-shot echo-planar-imaging sequence provided a 3.6 mm × 3.6 mm resolution over a 23-cm field of view. We accomplished T2^∗^ weighting from this sequence with an echo time of 30 ms and flip angle of 90°. We achieved whole brain coverage with a repetition time of 2000 ms allowing whole brain coverage with 24 slices of 6 mm thickness acquired in an interleaved manner without a gap between slices. Each woman provided 171 volumes of T2^∗^ functional data during a 5 min, 42 s scan. To allow an anatomical reference to the functional volumes and for voxel based morphometry and cortical thickness analysis, we acquired a 3-dimensional T1 magnetization-prepared rapid gradient echo (MPRAGE) volumetric scan with 3.44 ms echo time, 2250 ms repetition time, 900 ms TI, flip angle 9°, 96 slices, slice thickness 1.5 mm and 0.9 × 0.9-mm in-plane resolution over a 23-cm field of view.

### Circulating Sex Hormones

Clinical lab staff drew twenty milliliters of blood before each imaging session. To verify cycle phase and assess hormone levels, the blood samples were assayed with radioimmunoassay for estradiol, progesterone and free testosterone and enzyme-linked radio immunosorbent assay for LH and follicle stimulating hormone (FSH) (Veldhuijzen et al., 2013). Hormone assays were done at Johns Hopkins Medical Institute ICTR Clinical Research Core Laboratory. For estradiol, the intra-assay coefficient of variation was 4.2% and inter-assay coefficient of variation was 6.0%. For progesterone, the intra-assay coefficient of variation was 7.2% and inter-assay coefficient of variation was 9.0%. The limit of detection for estradiol was 2.2 pg/ml and for progesterone was 0.05 ng/ml.

### Electrical Stimulation

We delivered 20 Hz electrical stimuli to the left foot dorsum with 2 by 2 inch electrodes that passed a symmetrical biphasic pulse with a pulse width of 200 μs via a Empi 300PV Neuromuscular Stimulator (Empi, Clear Lake, SD, United States). For each participant, we determined a stimulus intensity for a painful stimulus, which they rated about 60 on a 0 to 100 visual analog scale (VAS) for pain corresponding to a moderate pain intensity. We determined the specific electrical stimulus intensity required to evoke a moderate pain intensity before each MRI session. This allowed us to match pain intensity across participants. Participants rated their pain intensity and pain unpleasantness on a 0–100 VAS with anchors for “no pain”/“not at all unpleasant,” and “most intense pain imaginable”/”extremely unpleasant.” Additionally, we assessed each participant’s electrical detection threshold and electrical pain threshold at each session. We instructed each participant that she could signal to end the protocol for any reason. No participant chose to end the protocol before it was complete.

### Voxel Based Morphometry Analysis

To make a first estimate of the whole brain voxel-wise effect of menstrual cycle phase on potential gray matter changes we used voxel-based morphometry as implemented in VBM8 (version r435) using the longitudinal analysis option (Ashburner and Friston, 2000). After an initial alignment using DARTEL, the mean of the realigned structural volumes was calculated (mean) and used as reference image in the subsequent realignment (Ashburner, 2007). We then bias corrected the realigned volumes to correct for signal in homogeneities with regard to the reference structural volume. We estimated spatial normalization parameters using the structural segmentations of the mean volume into gray matter, white matter, cerebral spinal fluid. We applied these normalization parameters to segmentations of the bias-corrected structural volumes. The resulting normalized segmentations are then again realigned.

### Freesurfer Cortical Thickness and White Matter Volume Analysis

The MPRAGE structural volumes were processed to remove all the skull and extra-cerebral tissue using afni’s 3dSkullStrip with options to maintain the original intensity of the volumes and push the strip mask to the edge of the brain. Since the primary measure of gray matter change in VBM is amount of change needed to morph an individual’s brain to a template, there are no standardized measurements created from the images (Ashburner, 2007). In order to obtain scalar measures of relative gray matter volume using an independent method, we implemented a cortical thickness analysis using Freesurfer. Each method has particular susceptibilities to T1 signal noise and methodological variability, therefore we only consider results which are statistically significant from both analyses on a whole-brain basis (Rajagopalan and Pioro, 2015; Chung et al., 2017). After skull-stripping, structural volumes for each subject for each session were processed using recon-all from Freesurfer version 5.1.0. Cortical thickness measures for Brodmann areas and cortical sulcal and gyral parcellations were tabulated and extracted for analysis in R 3.6.1 (Fischl et al., 2008; Destrieux et al., 2010). Following the regions found to be significantly associated with cycle phase during the VBM analysis, we examined white matter volume in the region of the left parietal lobe and gray matter thickness in the interparietal sulcus and transverse parietal sulcus. An additional hypothesis regarding somatosensory sensitivity being associated with menstrual cycle phase led us to analyze the menstrual cycle effects on both left and right BA1, BA2, BA3a, and BA3b analyzed within the same linear mixed model. The mixed model was specified to isolate the main effect of menstrual cycle phase from the known effects of Brodmann area and hemispheric asymmetry on cortical thickness. Specifically, fixed effects were modeled as (cycle phase + Brodmann area ^∗^ hemisphere) with random effects nested within subject to specify a repeated measures model (Galecki and Burzykowski, 2013). To display overall effects of menstrual cycle phase, cortical thickness was averaged over Brodmann area and hemisphere for each level of cycle phase. Finally, we tested left and right hemisphere as well as whole brain cortical gray matter and white matter volume as well as subcortical gray matter volume, whole brain gray matter volume and CSF volume for cycle phase effects. We modeled random effects as a function of session order nested within subject to specify a repeated measures model accounting for session order (Galecki and Burzykowski, 2013). Finally, each model was additionally tested with model weights for the fixed effect factor as a power function (varPower) of the phase order. As an additional control we report supplemental results modeling the weighting of the phase factor as a power function of the day of the menstrual cycle the session took place on for each subject. We tested all contrasts for each significant phase effect with the Holm-Sidak correction for multiple comparisons which controls family wise error. We display results as the estimated marginal means and standard error derived from the described linear mixed model (LMM). We use the R function anova to provide F-values for ease of interpretation of overall model significance. These estimations are likely conservative. The individual contrasts and corrected contrasts are the most accurate test of effect. Effect sizes were calculated from the raw data using Cohen’s d. To evaluate the effects of estradiol and progesterone on cortical thickness and white matter volume metrics we created trivariate and bivariate correlation model in the R package ppcor with a *post-hoc* correction for repeated measure adjustment of sample size (Kim, 2015; Bakdash and Marusich, 2017). We created zero-order correlation plots with ggscatter in R.

### Functional MRI Analysis

We analyzed functional imaging data with Analysis of Functional NeuroImages^1^. One of the 14 participants included in the cortical thickness and VBM analysis was excluded from the functional analysis because the resting state scan was not acquired during one session due to an equipment failure. We removed the first 4 volumes from the functional scan series to allow for signal equilibration. We then used afni_proc.py to generate automated scripts to process the resting functional MRI scans. We used 3dTshift for slice-timing correction, 3dDespike suppress spikes in the time voxel time series, and align_epi_anat.py to align each anatomical volume to the first functional volume of the scan series. We warped each anatomical volume to the Talairach normalized icbm452 volume template. We used 3dvolreg to motion correct each sessions’ time series. We censored functional volumes that were displaced more than 1.8 mm in Euclidean space. There were no censored volumes. A supplemental analysis showed only 2 volume-to-volume displacements that were greater than 0.9 mm, out of the 8,632 possible volume-to-volume displacements [13 participants × 4 sessions × (167 volumes-1)]. Once aligned, each structural volume was segmented into cerebral spinal fluid (CSF), gray matter (GM), and white matter (WM) segments using 3dSeg. These masks were projected into functional space (3.5 mm^3^ voxels) and the CSF and WM masks were eroded. The slice time corrected, despiked and registered functional time series were spatially blurred using an 8-mm full-width, half-maximum Gaussian filter. This FWHM filter was consistent with grand average blur estimates (x, y, z) = (8.14 mm, 8.32 mm, 7.97 mm). Then we used 3dBandpass to remove constant, linear and quadratic trends from the time series and apply a bandpass filter between 0.008 and 0.1 Hz.

For the subject-level seed-driven functional connectivity (SDFC) analysis, the peak F-stat voxel of menstrual cycle phase effect on gray matter volume from the VBM analysis was used as the initial seed region [Left inferior parietal lobule (IPL) = −27, −50, 56]. SDFC analysis was derived by extracting time series from each resting state functional scan from the left and right IPL (±27, −50, 56) using a spherical seed with a 6-mm radius. We used both left and right seeds since previous studies have found cycle phase effects on gray matter metrics in both left and right IPL and right and left IPL are core nodes of the dorsal attention network (Buckner and Vincent, 2007; Protopopescu et al., 2008; Lisofsky et al., 2015). We created subject-level SDFC maps by regressing the time series for the left and right IPL to each functional time series while using WM and CSF time series as well as six demeaned motion parameters (x, y, z, roll, pitch, and yaw) and their first derivatives as baseline regressors of no interest. The resultant R2 maps were converted to R maps and then to Z maps using Fisher’s R-to-Z transformation.

For group-level analysis we used the Z-score maps in a linear mixed model implemented using afni’s 3dLME (Chen et al., 2013). The model was specified to generate mean FC maps for each phase as well as FC collapsed across the menstrual cycle as well as contrast maps of ovulatory > luteal, ovulatory > menstrual, ovulatory > follicular, follicular > luteal, and luteal > menstrual. We used 3dClustSim with an autocorrelation function estimated as the average from all functional time series across all menstrual cycle phases (the acf was estimated as 69.3% Gaussian, with other parameters of 4.67 and 14.10 generating an effective FWHM of about 12 mm) (Cox et al., 2017). These settings in 3dClustSim, which address widely publicized vulnerabilities to Type I error in afni’s analysis framework, determined a cluster-extent criterion of 643 mm^3^ for a *p*-value of 0.001 (Eklund et al., 2016). This threshold is more than twice the volume of the minimal suggested cluster volume for whole brain analyses (Woo et al., 2014). All maps were thresholded using these parameters. All brain images are depicted in radiologic convention, where the right hemisphere is on the left of the image, and vice versa.

### Statistical Analysis of Psychophysical Measures

We evaluated the effect of menstrual cycle phase on EDTs, pain intensity and pain unpleasantness ratings in an LMM setting menstrual phase as a control and specifically assessing contrasts for menstrual phase compared to follicular, ovulatory and luteal phases. For electrical detection threshold, a measure of somatosensory sensitivity, we proposed *a priori* contrasts of women being more sensitive during ovulation and follicular phases when compared to luteal and menstrual phases. We report these results with both Holm-Sidak corrected and uncorrected *p*-values. We justify these *a priori* contrasts by taking into account previous findings of enhanced sensitivity during ovulation and follicular phases in several sensory modalities including visual, olfactory, auditory, and somatosensory (Herren, 1933; Diespecker and Kolokotronis, 1971; Diamond et al., 1972; Parlee, 1983; Hummel et al., 1991; Guttridge, 1994; Krug et al., 2000; Caruso et al., 2001, 2003; Grillo et al., 2001; Derntl et al., 2013; Renfro and Hoffmann, 2013; Veldhuijzen et al., 2013). We modeled fixed effects as cycle phase with random effects of session order nested within subject to specify a repeated measures model using R accounting for session order (Galecki and Burzykowski, 2013). Finally, we augmented each model with model weights for the fixed effect factor as a power function (varPower) of the phase order. As an additional control we report supplemental results modeling the weighting of the phase factor as a power function of the day of the menstrual cycle the session took place on for each subject. We tested all contrasts for each significant phase effect with the Holm-Sidak correction for multiple comparisons which controls family wise error. We display results as the estimated marginal means and standard error derived from the described LMM. Effect sizes were calculated from the raw data using Cohen’s d. Zero-order correlations were calculated using pcor with a *post-hoc* correction for repeated measure adjustment of sample size (Kim, 2015; Bakdash and Marusich, 2017).

## Results

### Gray Matter Volume and Cortical Thickness Are Greatest in Menstrual Phase

**FIGURE 1:**
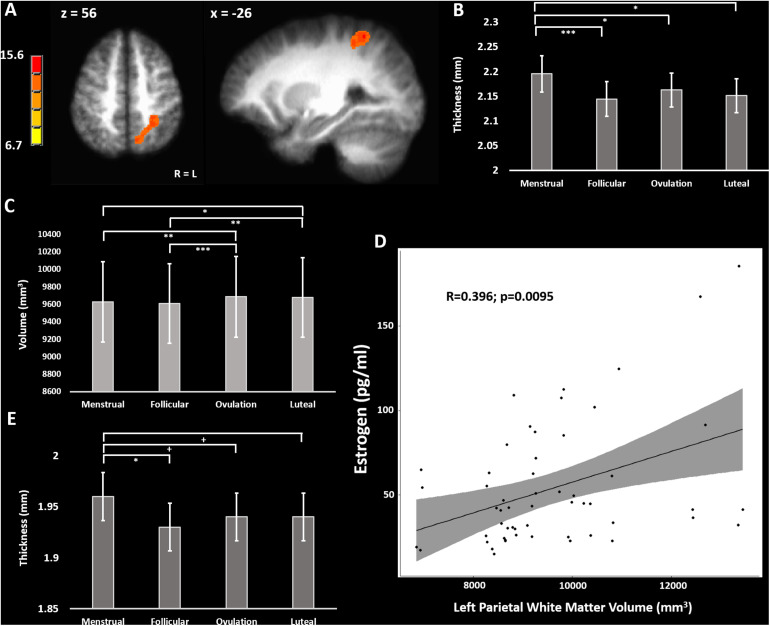
**(A)** F-map of significant variation in gray matter volume across the menstrual cycle. **(B)** Cortical thickness across the menstrual cycle in the left inferior parietal lobule. **(C)** White matter volume in the left parietal lobe across the menstrual cycle. **(D)** Positive correlation of left parietal white matter volume with blood estradiol concentrations across the menstrual cycle. **(E)** Cortical thickness across the menstrual cycle in the postcentral gyrus bilaterally (average over BA1, BA2, BA3a, and BA3b). + = 0.08 > *p* > 0.05, uncorrected; ^∗^ = *p* < 0.05; ^∗∗^ = *p* < 0.01; ^∗∗∗^ = *p* < 0.005, Holm-Sidak corrected.

A longitudinal voxel-based morphometry (VBM) analysis found one cluster which had a peak F-value of 15.6 in the left inferior parietal lobule (IPL) (TLRC coordinate = −27, −50, 56; volume = 2778 mm^3^), where the main effect of cycle phase significantly modulated gray matter volume (Figure 1A). Freesurfer parcellation and cortical thickness estimation showed that the left intraparietal and transverse parietal sulci were thickest during menstrual phase (main effect of cycle phase F-stat = 4.63; *p* = 0.037) with *post-hoc* comparisons significantly favoring thicker cortex during menstrual phase compared to follicular (z-stat = 3.45; Holm-Sidak-corrected *p* = 0.0034; Cohen’s *d* = 0.86) and luteal phase (z-stat = 2.92; Holm-Sidak-corrected *p* = 0.018; *d* = 0.67), and ovulatory phase (z-stat = 2.50; Holm-Sidak corrected *p* = 0.0496; *d* = 0.70) (Figure 1B). Furthermore, there was a main effect of cycle phase on white matter underlying the left IPL (*F* = 6.89; *p* = 0.0008) (Figure 1C). *Post-hoc* tests showed greater white matter volume during ovulatory compared to follicular (z-stat = 3.79; Holm-Sidak-corrected *p* = 0.0009; *d* = 0.81) or menstrual phase (z-stat = 3.08; Holm-Sidak-corrected *p* = 0.0082; *d* = 0.73) and greater white matter volume during luteal compared to follicular phase (z-stat = 3.23; Holm-Sidak-corrected *p* = 0.0061; *d* = 0.93) or menstrual phase (z-stat = 2.51; Holm-Sidak corrected *p* = 0.036; *d* = 0.87).

To determine the potential effects of estradiol and progesterone on left intraparietal and transverse parietal sulci cortical thickness we used a partial correlation model comparing cortical thickness to estradiol and progesterone, and simple correlation to estradiol and progesterone concentrations separately. No correlations of left parietal cortical thickness with estradiol (*R* < 0.153) or progesterone (*R* > −0.065) surpassed *R* = 0.153. We repeated this analysis for left parietal white matter volume. In the trivariate partial correlation model, we found no significant relationship between left parietal white matter volume and progesterone (*R* = 0.036; sample-size corrected *p* = 0.83), but a significant relationship between left parietal white matter volume and estradiol (*R* = 0.452; sample-size corrected *p* = 0.0022). There remained a moderate correlation between estradiol and left parietal white matter volume in the zero-order correlation [*R* = 0.479; sample-size corrected *p* = 0.0013 (without outlier: *R* = 0.396, *t*-stat = 2.73, sample-size corrected *p* = 0.0095)] (Figure 1D).

We sought to evaluate the effect of menstrual cycle on cortical thickness of the bilateral somatosensory cortex that may relate to the previously reported menstrual cycle effect on tactile and thermal sensory sensitivity as well as pressure pain sensitivity in this particular cohort (Herren, 1933; Kenshalo, 1966; Diespecker and Kolokotronis, 1971; Gescheider et al., 1984; Teepker et al., 2010; Veldhuijzen et al., 2013; Alves et al., 2017). We combined measurements from BA1, BA2, BA3a, and BA3b bilaterally into a linear mixed model isolating the effect of menstrual cycle phase from effects of Brodmann area and hemisphere (Fischl et al., 2008). The model revealed a significant main effect of menstrual cycle phase (*F* = 3.03; *p* = 0.029). *Post-hoc* corrected contrasts showed primary somatosensory cortex (S1) was thicker during menstrual phase compared to follicular phase (z-stat = 2.81; Holm-Sidak-corrected *p* = 0.030; *d* = 0.51). Trends for cortical thickness indicated S1 was also thicker during menstrual phase when compared to luteal (z-stat = 2.22; uncorrected *p* = 0.027; *d* = 0.36) and ovulatory phase (z-stat = 1.84; uncorrected *p* = 0.066; *d* = 0.30) did not survive Holm-Sidak correction (Figure 1E).

Control analyses of left and right hemisphere as well as whole brain cortical gray matter volume did not show a main effect of menstrual cycle phase (LH: F = 2.47, *p* = 0.077; RH: *F* = 0.88, *p* = 0.46; whole brain: *F* = 1.87, *p* = 0.15) (Table 1). Additional analyses of right hemisphere mean cortical thickness revealed no effect of menstrual cycle phase (*F* = 1.09, *p* = 0.36). In contrast, analysis of left hemisphere mean cortical thickness revealed a significant effect of menstrual cycle phase (*F* = 3.12, *p* = 0.037). *Post-hoc* contrasts revealed that mean cortical thickness in the left hemisphere of the cerebral cortex was greater during menstrual phase compared to luteal phase (z-stat = 2.73; Holm-Sidak-corrected *p* = 0.038; *d* = 0.82). Additionally, there was a trend for left hemisphere cortical thickness to be greater during menstrual phase compared to follicular phase (z-stat = 2.33; uncorrected *p* = 0.020; *d* = 0.57). The mean difference of the significant contrast was 0.02 mm [menstrual phase mean = 2.42 mm (95% CI 2.37–2.47 mm) versus luteal phase mean = 2.40 mm (95% CI 2.35–2.45 mm)].

**TABLE 1:** Summary of overall relationships of menstrual cycle phase, estradiol and progesterone with brain morphometry measures.

	Effects of menstrual cycle	Correlation with estradiol	Correlation with progesterone
**Whole Brain Cortical Gray Matter Volume**	No significant cycle phase effect	Not significant	Not significant
**Right Hemisphere Cortical Gray Matter Volume**	No significant cycle phase effect	Not significant	Not significant
**Left Hemisphere Cortical Gray Matter Volume**	No significant cycle phase effect	Not significant	Not significant
**Left Hemisphere Cortical Gray Matter Thickness**	Menstrual > Luteal* Menstrual > Follicular^+^	Not significant	Not significant
**Right Hemisphere Cortical Gray Matter Thickness**	No significant cycle phase effect	Not significant	Not significant
**Whole Brain White Matter Volume**	No significant cycle phase effect	Not Significant	Not significant
**Left Hemisphere White Matter Volume**	No significant cycle phase effect	Positive correlation*	Not significant
**Right Hemisphere White Matter Volume**	Follicular > Luteal* Ovulatory > Luteal^+^	Positive correlation*	Not significant
**Subcortical Gray Matter Volume**	No significant cycle phase effect	Not significant	Not significant
**Cerebrospinal Fluid Volume**	No significant cycle phase effect	Not significant	Not significant

*significant relationship after *post-hoc* correction for family wise errors. ^+^trend relationship after *post-hoc* correction for family wise errors.

To evaluate the effect of estradiol and progesterone concentrations on cortical gray matter thickness, we evaluated the partial correlation of left and right hemisphere cortical thickness, separately, to estradiol and progesterone and simple correlation of cortical thickness to estradiol and progesterone concentrations separately. No correlations of cortical thickness of the left or right hemisphere cortical gray matter thickness with estradiol (*R* < 0.155) or progesterone (*R* > −0.047) surpassed *R* = 0.155.

Finally, control analyses of left hemisphere as well as whole brain white matter volume did not show a main effect of menstrual cycle phase (LH: *F* = 1.21, *p* = 0.32; whole brain: *F* = 1.85, *p* = 0.15). In contrast, the linear mixed model (LMM) of right hemisphere white matter volume showed a main effect of menstrual cycle phase (RH: *F* = 3.69, *p* = 0.020). *Post-hoc* contrasts revealed that mean white matter volume in the right hemisphere was greater during follicular phase compared to luteal phase (z-stat = 3.09; Holm-Sidak-corrected *p* = 0.012; *d* = 0.15). Additionally, there was a trend for right hemisphere white matter volume to be greater during ovulatory phase compared to luteal phase (z-stat = 2.56; Holm-Sidak-corrected *p* = 0.053; *d* = 0.26).

We evaluated the partial correlation of right hemisphere white matter volume to estradiol and progesterone as well as the simple correlation of white matter volume to estradiol and progesterone concentrations separately. Whereas no relationships between right hemisphere white matter volume and progesterone concentrations were significant (*R* = 0.086, *p* = 0.586), a positive relationship between right hemisphere white matter volume and estradiol was found in both the partial correlation model controlling for progesterone (*R* = 0.409, sample size corrected *p* = 0.0079), and in the simple correlation with estradiol concentration [*R* = 0.414, t-stat = 2.88, sample size corrected *p* = 0.0064 (without outlier: *R* = 0.355, t-stat = 2.40, sample size corrected *p* = 0.021)]. Given this evidence of a positive relationship of estradiol with right hemisphere white matter volume, we evaluated this relationship with left hemisphere white matter volume. In this case, a positive relationship between left hemisphere white matter volume and estradiol was found in both the partial correlation model controlling for progesterone (*R* = 0.420, t-stat = 2.93, sample size corrected *p* = 0.0056) and in the zero-order correlation with estradiol concentration [*R* = 0.427, t-stat = 2.99, sample size corrected *p* = 0.0048 (without outlier: *R* = 0.363, t-stat = 2.46, sample size corrected *p* = 0.018)].

Finally, there was no main effect of phase found for subcortical gray matter volume (*F* = 0.11, *p* = 0.95), whole brain gray matter volume (*F* = 1.46, *p* = 0.24), or CSF volume (*F* = 0.82, *p* = 0.49). These brain-wide morphometric relationships are summarized in Table 1.

### Menstrual Cycle Effects Upon Electrical Detection and Pain Sensitivity

**FIGURE 2:**
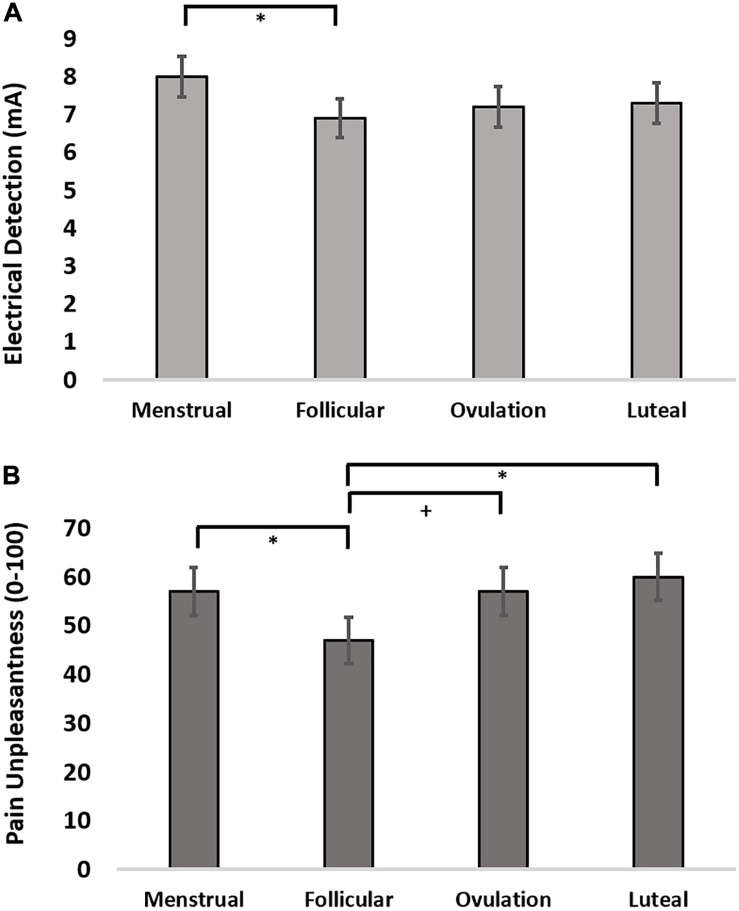
**(A)** Electrical detection threshold across the menstrual cycle. **(B)** Pain unpleasantness ratings in response to suprathreshold electrical pain stimuli across the menstrual cycle. + = 0.08 > *p* > 0.05, uncorrected; ^∗^ = *p* < 0.05, Holm-Sidak corrected.

The finding that cortical thickness was greatest during menstrual phase led us to evaluate the effect of menstrual cycle on electrical detection thresholds (EDT), pain intensity and pain unpleasantness reports to suprathreshold electrical stimulation. We evaluated the effect of menstrual cycle phase in an LMM setting menstrual phase as a control and specifically assessing contrasts for menstrual phase compared to follicular, ovulatory, and luteal phases. The LMM for menstrual cycle effect on EDTs revealed that women were significantly more sensitive to detecting electrical stimulation during follicular phase (z-stat = 2.16; uncorrected *p* = 0.031; *d* = 0.59) and trended toward greater sensitivity during ovulatory phase (z-stat = 1.79; uncorrected *p* = 0.074; *d* = 0.36) (Figure 2A). Pain intensity self-report in response to suprathreshold electrical stimuli did not show an effect of menstrual cycle phase (*F* = 1.56, *p* = 0.21; z-stat < 1.86, *p* > 0.063). Further, there was no menstrual cycle phase effect on electrical pain thresholds (*F* = 1.06, *p* = 0.38; t-stat < 1.64, *p* < 0.10). In contrast to pain sensitivity measures, pain unpleasantness ratings were greater during menstrual phase compared to follicular phase (z-stat = 2.34; uncorrected *p* = 0.019; *d* = 0.61) (Figure 2B). Exploratory contrasts, supported by a significant main effect of menstrual cycle phase on pain unpleasantness rating (*F* = 3.00; *p* = 0.043), revealed pain unpleasantness ratings during follicular phase were lower compared to luteal (z-stat = 2.74; Holm-Sidak-corrected *p* = 0.037; *d* = 0.76) and trended lower compared to ratings reported during ovulatory phase (z-stat = 1.92; uncorrected *p* = 0.054; *d* = 0.44).

No simple or partial correlation test relationships between estradiol (−0.219 < *R* < 0.056) or progesterone (−0.0168 < *R* < 0.231) and quantitative sensory test measures were significant.

### Maximal Effects of Menstrual Cycle Phase on Gray Matter Volume Correspond With a Central Node of the Dorsal Attention Network

**FIGURE 3:**
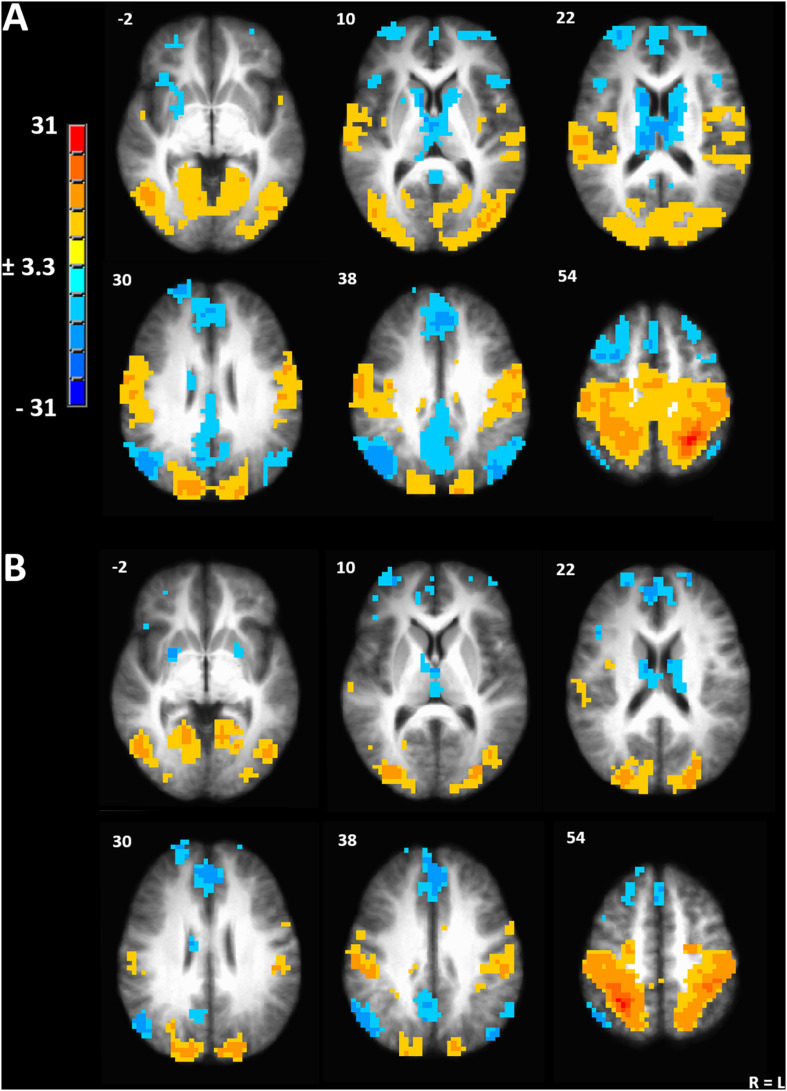
**(A)** Seed-driven network from the left inferior parietal lobule. **(B)** Seed-driven network from the right inferior parietal lobule. Cluster-extent corrected at a *p*-value of 0.001 and cluster size of 643 mm^3^.

We used the peak voxel in the left IPL (TLRC coordinate = −27, −50, 54) from the menstrual cycle phase effect on gray matter volume to drive seed-based functional connectivity analysis (Figure 3A). Both the resulting network and the mirrored network derived from the right IPL (TLRC coordinate = 27, −50, 54) closely resembled the dorsal attention network (DAN), which involves the frontal eye fields, intraparietal sulcus and bilateral visual cortex [Figure 3B; (Fox et al., 2006; Laird et al., 2011)].

### Dorsal Attention Network Functional Connectivity Between Right and Left IPL and Between Left IPL and Visual Cortex Peaks During Ovulatory Phase

**FIGURE 4:**
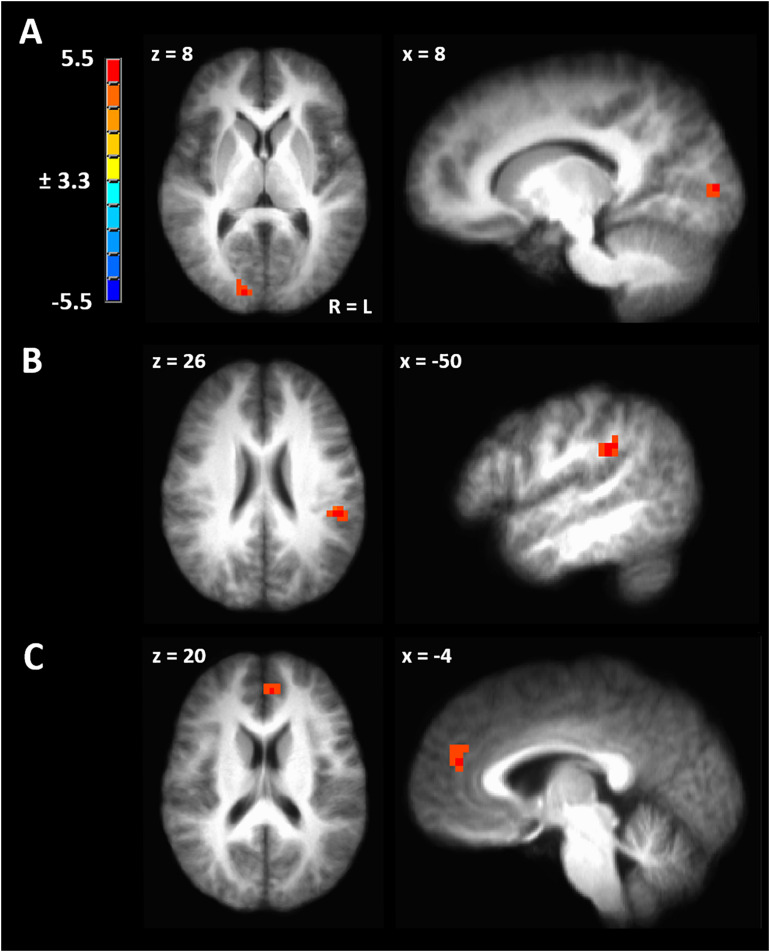
**(A)** Significant cluster from functional connectivity driven from left inferior parietal lobule from the contrast of ovulatory phase > luteal phase. **(B)** Significant cluster from functional connectivity driven from right inferior parietal lobule from the contrast of ovulatory phase > follicular phase. **(C)** Significant cluster from functional connectivity driven from right inferior parietal lobule from the contrast of luteal > menstrual phase. Cluster-extent corrected at a *p*-value of 0.001 and cluster size of 643 mm^3^.

To evaluate the effect of menstrual cycle phase on functional connectivity, we contrasted all pairwise phase comparisons from a linear mixed model (Chen et al., 2013). After a strict cluster-extent-correction, functional connectivity was significantly greater during ovulatory compared to luteal phase between the left IPL seed and the right visual cortex (peak TLRC coordinate = 12, −88, 8; peak voxel t-stat = 4.65; peak voxel Cohen’s *d* = 0.98; volume = 686 mm^3^) (Figure 4A). Ovulatory phase functional connectivity between left IPL and right visual cortex was also greater when compared to FC during menstrual phase, but this cluster did not pass our strict cluster extent criteria (peak TLRC coordinate = 16, −82, 7; peak voxel t-stat = 3.50; peak voxel Cohen’s *d* = 1.07; volume = 172 mm^3^) (Supplementary Figure 1A).

Seed-driven FC from the right IPL to the left IPL was significantly greater during ovulatory compared to follicular phase (peak TLRC coordinate = −50, −32, 32; peak voxel t-stat = 5.33; peak voxel Cohen’s *d* = 1.60; volume = 1200 mm^3^) (Figure 4B). Further, ovulatory phase FC was also greater compared to FC during menstrual (peak TLRC coordinate = −54, −33, 31; peak voxel t-stat = 3.12; peak voxel Cohen’s *d* = 0.70; volume = 172 mm^3^) and luteal phase (peak TLRC coordinate = −47, −36, 31; peak voxel t-stat = 3.13; peak voxel Cohen’s *d* = 0.93; volume = 172 mm^3^) but neither of these clusters passed our cluster extent criteria (Supplementary Figures 1B,C).

Finally, seed-driven FC was greater during luteal phase compared to menstrual phase from the right IPL seed to left medial prefrontal cortex (BA9) (peak TLRC coordinate = −5, 44, 20; peak voxel t-stat = 4.58; peak voxel Cohen’s *d* = 1.50; volume = 858 mm^3^) (Figure 4C).

## Discussion

We hypothesized significant variation in gray matter volume and cortical thickness across the menstrual cycle in healthy human females. In a voxel-based morphometry analysis of the whole brain we found a significant main effect of phase involving the left IPL (Figure 1A). Cortical gray matter thickness analysis of the left IPL found that the effect of menstrual cycle phase was driven by increased cortical thickness during the menstrual phase compared to follicular or luteal phase as well as a trend for greater thickness during menstrual compared to ovulatory phase (Figure 1B). A similar pattern was found bilaterally in the postcentral gyrus (Figure 1E). Potentially related, women in this study were more sensitive to detection of electrical stimuli during follicular phase compared to menstrual phase (Figure 2A). Pain unpleasantness ratings to fixed intensity painful electrical stimuli were lowest during follicular phase compared to menstrual, ovulatory, or luteal phase (Figure 2B). The volume of white matter in the left parietal lobe was larger during ovulatory phase compared to follicular and menstrual phases and was greater during luteal compared to follicular and menstrual phase (Figure 1C). We also hypothesized that the region of significant gray matter cycle effects would significantly fluctuate in functional connectivity (FC) in resting state MRI scans. In testing our hypothesis, we used the left IPL as a seed for seed-driven FC. Supporting our hypothesis, we found FC between left IPL and right visual cortex was greater in ovulatory compared to luteal phase with a trend toward greater FC in ovulatory compared to menstrual phase (Figure 4A). Using an IPL seed in the right hemisphere, we found significantly greater FC between right IPL and left IPL during ovulatory compared to follicular phase with a trend toward greater FC during ovulatory compared to menstrual or luteal phase (Figure 4B). Finally, FC between right IPL and left medial prefrontal cortex (BA9) was greater during luteal phase compared to menstrual phase (Figure 4C). These findings support the hypothesis that natural cycle effects occur in brain matter volume (both gray and white matter) and in functional connectivity which indicates hormonal influence on specific brain regions, particularly the IPL.

### Functional Connectivity of Dorsal Attention Network, Sensory Sensitivity and Menstrual Cycle Influenced Tasks

The IPL is a core node in the dorsal attention network, also frequently called the task positive network, which is considered to be responsible for top-down target-directed and sustained attention (Pardo et al., 1991; Corbetta and Shulman, 2002; Fox et al., 2006). We found enhanced FC between the right and left IPL and between the left IPL and the right visual cortex during the ovulatory phase. Peak sensitivity to a broad range of stimuli during ovulatory phase, would likely be evolutionarily advantageous in detection of potential mates and determination of mate fitness (Jones et al., 2019). Numerous studies have been dedicated to testing the hypothesis that male faces communicate potential mate fitness or signs of testosterone or simply masculinity, which women are most sensitive to during periods of high fertility (Penton-Voak et al., 1999; Gildersleeve et al., 2014a,b; Harris et al., 2014; Wood and Carden, 2014). These findings are generally mixed, specific for short-term mating contexts, and controversial, with several studies not finding the effect of a preference for masculine or symmetric faces during ovulatory phase. Sensory perception, particularly visual and olfactory perception, are frequently reported as being most sensitive during follicular or ovulatory phase; while preferences for masculine and feminine faces are particular to recently developed urbanized cultures (Scott et al., 2014; Jones et al., 2018). It would be more parsimonious to hypothesize that the enhanced FC between critical nodes of the dorsal attention network drive an increase in a broad range of perceptual sensitivity particularly for evolutionary salient stimuli (Gould and Lewontin, 1979; Krug et al., 2000; Pigliucci and Kaplan, 2000). The finding that race bias tracks conception risk across the menstrual cycle further supports the view that changes in perceptual sensitivity are co-opted to serve novel cultural constructs relevant to mating strategies rather than specific preferences for masculine features being selected for Navarrete et al. (2009). Furthermore, the proximate explanation of enhanced perceptual sensitivity and the ultimate, functional explanation of preference for certain facial features during ovulation can coexist within an evolutionary framework (Scott-Phillips et al., 2011). Enhanced neural plasticity induced by estradiol in the hippocampus, prefrontal and parietal cortex during ovulatory phase would enhance adaptivity of females to changing cultural conceptions of fitness in potential mates (Hao et al., 2006; Rupp et al., 2009a,b; Wallen and Rupp, 2010; Wang et al., 2010). It must be noted that we did not replicate reports of amygdala or hippocampal plasticity across the menstrual cycle in this cohort likely because of a lack of power (Ossewaarde et al., 2013; Engman et al., 2018). Therefore, it should not be concluded that cycle-related neural plasticity in the limbic system is absent, or even less prominent than that occurring in the dorsal attention network. Finally, hormone-related fluctuations of neural plasticity would not only affect potential mechanisms of mate selection, but would extend to influences on cognition, emotion processing, motivation and sensitivity to disease (Kibler et al., 2005; Sundstrom Poromaa and Gingnell, 2014; Albert et al., 2015; Herzog et al., 2015; Pilarczyk et al., 2019).

### Gray Matter Morphology and Sensory Sensitivity Are Maximally Divergent During Follicular Phase

In this study, we found that cortical gray matter thickness in the primary somatosensory cortex was thinnest during follicular phase, when women showed greatest electrical detection sensitivity. A previous study found a change in whole brain volumes with the menstrual cycle, therefore we evaluated whether the change in left IPL and bilateral somatosensory cortex cortical thickness was part of a whole brain volume change (Hagemann et al., 2011). We did not find any significant effects of menstrual cycle phase on total brain volume. Notably, we were unable to replicate the previous reports of menstrual cycle phase on hippocampal volume, after requiring significant effects in both cortical thickness analysis and VBM and applying a conservative cluster-size correction to limit the false discovery rate to an acceptable threshold (Protopopescu et al., 2008; Lisofsky et al., 2015). The change in cortical thickness and gray matter volume in the parietal cortex across the menstrual cycle suggests enhanced plasticity associated with estradiol and progesterone cycling in the brain (Lord et al., 2010; Thimm et al., 2014; Lisofsky et al., 2015; Motley et al., 2018).

During the follicular phase, pain unpleasantness ratings were lowest compared to other cycle phases in response to painful electrical stimuli while detection sensitivity to non-painful cutaneous electrical stimuli was greatest. Previous studies have reported heightened sensitivity to sensory stimuli including auditory, olfactory, tactile, cool and visual stimuli during the follicular or ovulatory phase (Diamond et al., 1972; Parlee, 1983; Symons et al., 1990; Hummel et al., 1991; Guttridge, 1994; Caruso et al., 2001, 2003; Grillo et al., 2001; Navarrete-Palacios et al., 2003; Derntl et al., 2013; Renfro and Hoffmann, 2013; Alves et al., 2017). Previously reported effects of ovarian hormones showed that healthy females of reproductive age on anti-androgenic progestin oral contraceptives when compared to normally cycling controls or females on androgenic progestins have increased gray matter volume in the bilateral fusiform face area and parahippocampal place area coupled with enhanced performance in a face recognition performance task compared to women during the menstrual phase of their cycle (Pletzer et al., 2015). In this study we found reduced cortical thickness associated with increased sensitivity to non-painful cutaneous electrical stimulation. A recent study evaluating cortical thickness differences between healthy males and females found that, while there was large overlap in cortical thickness distributions between the sexes, once cortical thickness was corrected for intracranial volume, women had thicker cortical regions in the bilateral superior and inferior parietal lobules, bilateral paracentral lobules and left postcentral gyrus (Ritchie et al., 2018). The change in cortical thickness in the parietal cortex across the menstrual cycle suggests enhanced plasticity associated with estradiol and progesterone cycling in the brain may potentially underlie this sex difference (Protopopescu et al., 2008; Pletzer et al., 2010; Lisofsky et al., 2015; De Bondt et al., 2016). With relatively few studies comparing sensory discrimination or task performance in association with women taking various oral contraceptives and during different phases of the menstrual cycle, the relationship between hormone-associated changes in cortical thickness and perceptual sensitivity or task performance remains unclear.

### Potential Cellular Mechanisms of Morphological Alteration in Gray Matter

Golgi impregnation studies in rats and non-human primates have demonstrated that estradiol enhances the formation of dendritic spines and synaptic densities in the hippocampus, prefrontal and parietal cortices (Woolley et al., 1990; Woolley and McEwen, 1992, 1993; Choi et al., 2003; Hao et al., 2003, 2006; Chen et al., 2009; Hara et al., 2016, 2018; Motley et al., 2018). The marginal increase in cortical thickness we found at the ovulatory phase may be driven by estradiol effects at that level. The large increase in cortical thickness captured during the menstrual phase is less likely explained by that mechanism. However, if the withdrawal of estradiol and progesterone is associated with the trigging of process of reduction in dendritic spine and synapse density, it may be that there is an increase in astroglial cellular activity local to brain areas experiencing large-scale pruning of nascent synaptic connections (May et al., 2007; Pfeiffer et al., 2016). Other studies have found evidence that skill learning in healthy humans increases cortical thickness, while memory performance positively correlates with gray matter volume (Draganski et al., 2004, 2006; Schmidt-Wilcke et al., 2009). This suggests that experience-induced neural plasticity does indeed induce increases in cortical thickness. Further studies should examine the exact cellular mechanism underlying the increase in cortical thickness captured at the menstrual phase.

Estradiol has been known to accelerate developmental myelination for more than 50 years (Curry and Heim, 1966). Estradiol promotes neuroprotection of oligodendrocytes and Schwann cells in cell culture and opposes demyelination *in vivo* in rodent models of hypoxia and demyelination by cuprizone (Gerstner et al., 2007; Acs et al., 2009). Estrogen replacement therapy in adult ovariectomized rats furthermore increases the degree and volume of myelination (He et al., 2018). Both estradiol and progesterone promote expression of myelin basic protein in oligodendrocytes and Schwann cells (Jung-Testas et al., 1994). Membrane-bound estrogen receptors (mER) α and β are present in oligodendrocytes, mediating activation of p42/44 mitogen-activated protein kinase (MAPK) and Akt through phosphorylation (Hirahara et al., 2009). Additionally, mER has been found in the myelin product of oligodendrocytes (Arvanitis et al., 2004). These non-genomic receptors in oligodendrocytes, of course, do not rule out the more traditional role of estrogen receptor acting as a transcription factor. In line with these previous findings, oligodendrocyte derived changes in myelination across the menstrual cycle may potentially underlie the positive correlation we found throughout the menstrual cycle between white matter volume and estradiol concentration that was most prominent in the left inferior parietal lobule (Figure 1D). These estradiol-associated menstrual cycle change in white matter volume are consistent with past observations in rats and across adolescent women using diffusion weighted imaging and structural volumetric measurements (Prayer et al., 1997; Herting et al., 2012).

### Limitations

The evidence in this report was subjected to rigorous statistical criteria, but for menstrual cycle studies this study had a relatively small sample size (*n* = 14 gray matter; *n* = 13 functional connectivity). For example, recent studies evaluating effective sample sizes for potential facial masculinity preference among normally cycling women required sample sizes between 55 and 71 for within subjects designs with confirmed ovulation (Jones et al., 2019). Additionally, this particular cohort of participants were only tested for electrocutaneous detection and pain perception. Therefore, these results should be considered preliminary.

Furthermore, extensive research in animal models demonstrates the importance of local neurosteroidogenesis in the brain (Rudolph et al., 2016). There is evidence of regional variation in aromatase concentrations in various areas of the human brain and neuroactive steroids are clearly locally produced and metabolized depending on local concentration of metabolic enzymes, substrates, and products (Takahashi et al., 2014). Furthermore, sex hormone binding globulin local concentrations keep readily releasable pools of steroids available and difficult to measure (Södergård and Bäckström, 1987). However, it is clear that there is a relationship between blood serum and cerebral spinal fluid concentrations of neuroactive steroids and steroids in blood circulation clearly cross the blood brain barrier in both humans and animal models as demonstrated by 16a-18F-Fluoro-17b-Estradiol positron emission tomography (Khayum et al., 2014; Kurland et al., 2020). Furthermore, blood serum progesterone and estradiol correlations with human cerebrospinal fluid are relatively rare with one notable study finding positive correlations of 0.731 for estradiol and 0.913 for progesterone (Bäckström et al., 1976). These correlations between blood plasma and cerebral cortical tissue in rats was 0.86 for estradiol and 0.46 for progesterone (Caruso et al., 2013).

### Conclusion and Future Research

We found a significant effect of cycle phase on gray matter volume and thickness in the left IPL, which was greatest during menstrual phase. Further, we found that FC between left IPL and right IPL as well as left IPL and right visual cortex was enhanced during the ovulatory phase. These findings are associated with previous findings of enhanced perceptual sensitivity during ovulatory phase across multiple sensory domains. Future research should replicate and extend these results. These significant fluctuations of neural plasticity and activity across the menstrual cycle are likely a sexually selected feature which may have a role in commonly experienced menstrual cycle-associated disorders such as premenstrual dysphoric disorder, catamenial epilepsy and migraine disorder (Hattemer et al., 2007; Martin and Lipton, 2008; Vincent et al., 2011; Reddy, 2013; Tu et al., 2013).

## Data Availability Statement

The raw data supporting the conclusions of this article will be made available by the authors, without undue reservation.

## Ethics Statement

The studies involving human participants were reviewed and approved by University of Maryland, Baltimore (UMB) Institutional Review Board for the Protection of Human Subjects. The patients/participants provided their written informed consent to participate in this study.

## Author Contributions

TM contributed to analyze data, data interpretation, preparing figures, draft and revise manuscript, and final approval. DV contributed to design, collect data, data interpretation, revise manuscript, and final approval. MK contributed to collect data, analyze data, preparing figures, and final approval. RG contributed to design, data interpretation, and final approval. JG contributed to design, collect data, data interpretation, revise manuscript, and final approval. All authors contributed to the article and approved the submitted version.

## Conflict of Interest

The authors declare that the research was conducted in the absence of any commercial or financial relationships that could be construed as a potential conflict of interest.

## Footnotes

1 http://afni.nimh.nih.gov
