# Supplementary material for: Menstrual Cycle Variations in Gray Matter Volume, White Matter Volume and Functional Connectivity: Critical Impact on Parietal Lobe
Source: Front Neurosci. 2020 Dec 22;14:594588. doi: 10.3389/fnins.2020.594588 (PMC7783210; doi:10.3389/fnins.2020.594588)
Supplement: Supplementary file 1 [file Image_1.pdf]

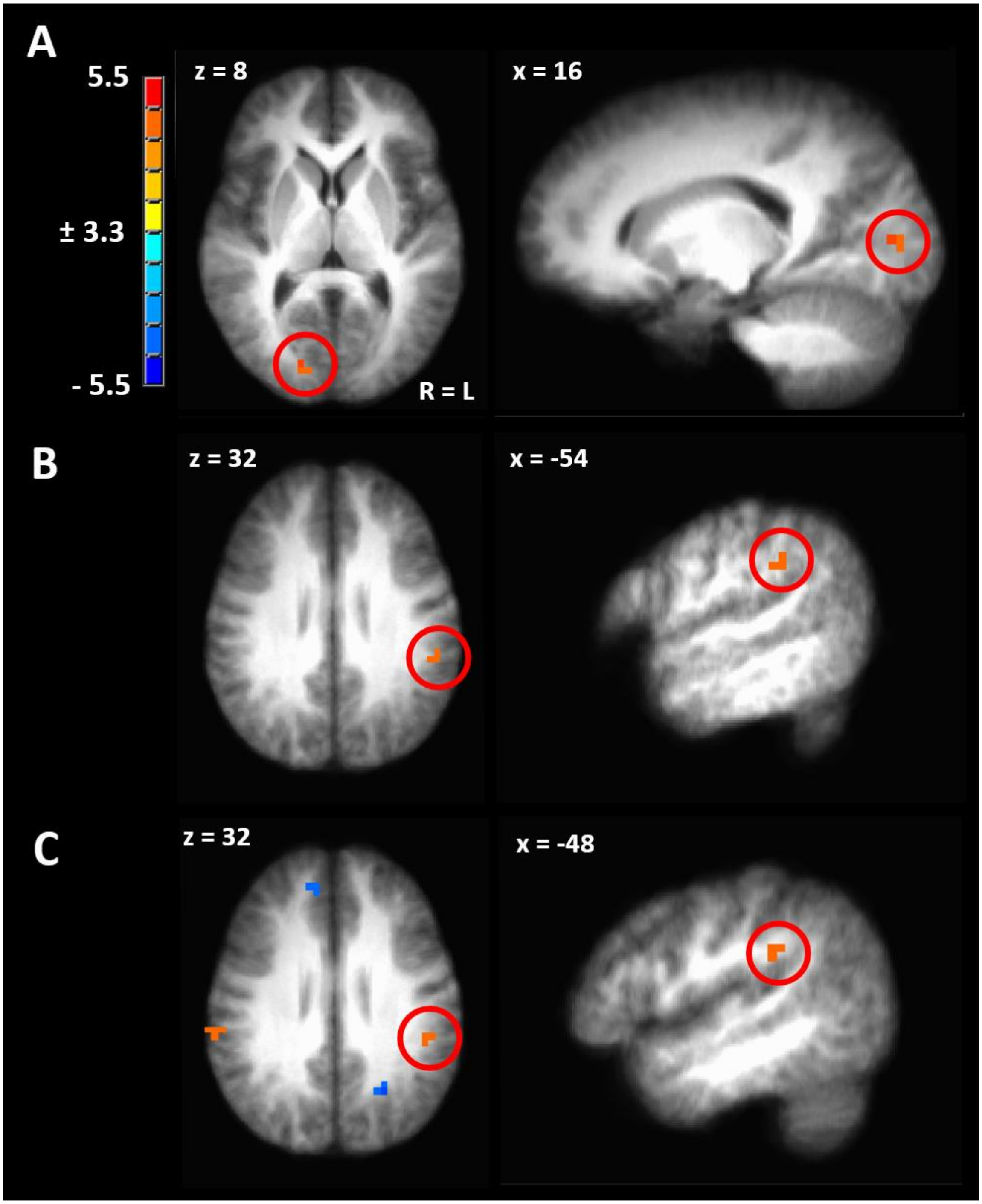

Supplemental Figure 1. A) Significant cluster from functional connectivity driven from left inferior parietal lobule from the contrast of ovulatory phase > menstrual phase. **B)** Significant cluster from functional connectivity driven from right inferior parietal lobule from the contrast of ovulatory phase > menstrual phase. **C)** Significant cluster from functional connectivity driven from right inferior parietal lobule from the contrast of ovulatory phase > luteal phase. While these maps were thresholded at $p<0.001$, no clusters passed cluster-extent correction for multiple comparisons.
